# The cell of origin dictates the temporal course of neurofibromatosis-1 (*Nf1*) low-grade glioma formation

**DOI:** 10.18632/oncotarget.17589

**Published:** 2017-05-03

**Authors:** Anne C Solga, Joseph A Toonen, Yuan Pan, Patrick J Cimino, Yu Ma, Guillaume A Castillon, Scott M Gianino, Mark H Ellisman, Da Yong Lee, David H Gutmann

**Affiliations:** ^1^ Department of Neurology and Washington University School of Medicine, St. Louis MO, USA; ^2^ Pathology and Immunology, Washington University School of Medicine, St. Louis MO, USA; ^3^ National Center for Microscopy and Imaging Research, University of California, San Diego CA, USA

**Keywords:** astrocytoma, pediatric brain tumor, OPC, tumorigenesis, NF1

## Abstract

Low-grade gliomas are one of the most common brain tumors in children, where they frequently form within the optic pathway (optic pathway gliomas; OPGs). Since many OPGs occur in the context of the Neurofibromatosis Type 1 (NF1) cancer predisposition syndrome, we have previously employed *Nf1* genetically-engineered mouse (GEM) strains to study the pathogenesis of these low-grade glial neoplasms. In the light of the finding that human and mouse low-grade gliomas are composed of Olig2^+^ cells and that Olig2^+^ oligodendrocyte precursor cells (OPCs) give rise to murine high-grade gliomas, we sought to determine whether Olig2^+^ OPCs could be tumor-initiating cells for *Nf1* optic glioma. Similar to the GFAP-Cre transgenic strain previously employed to generate *Nf1* optic gliomas, Olig2^+^ cells also give rise to astrocytes in the murine optic nerve *in vivo*. However, in contrast to the GFAP-Cre strain where somatic *Nf1* inactivation in embryonic neural progenitor/stem cells (*Nf1^flox/mut^*; GFAP-Cre mice) results in optic gliomas by 3 months of age *in vivo*, mice with *Nf1* gene inactivation in Olig2^+^ OPCs (*Nf1^flox/mut^*; Olig2-Cre mice) do not form optic gliomas until 6 months of age. These distinct patterns of glioma latency do not reflect differences in the timing or brain location of somatic *Nf1* loss. Instead, they most likely reflect the cell of origin, as somatic *Nf1* loss in CD133*^+^* neural progenitor/stem cells during late embryogenesis results in optic gliomas at 3 months of age. Collectively, these data demonstrate that the cell of origin dictates the time to tumorigenesis in murine optic glioma.

## INTRODUCTION

Low-grade glial neoplasms (pilocytic astrocytoma; PA) account for the majority of brain tumors in children between the ages of five and fifteen. In contrast to adult high-grade gliomas, low-grade gliomas in children typically arise in cerebellum, brainstem and optic pathway. While the majority of PAs developing within the cerebellum harbor *BRAF* gene rearrangements [1], one of the most common genetic etiologies for optic pathway gliomas (OPGs) is the neurofibromatosis type 1 (NF1) inherited cancer syndrome. Unlike their sporadic counterparts, these NF1-OPGs result from bi-allelic inactivation of the *NF1* tumor suppressor gene [2–4]. However, similar to PAs arising in other brain locations, NF1-OPGs contain low proliferative indices (< 1%), and harbor both glial fibrillary acidic protein (GFAP)- and Olig2-immunoreactive cells [5].

In the context of NF1, 15–20% of individuals will develop an OPG, which can arise at any age during childhood, ranging from infancy [6] to the early teens [7]. Moreover, there is a considerable spectrum of anatomic and clinical heterogeneity. As such, NF1-OPGs can form anywhere along the optic pathway from the optic nerves to the optic radiations, and only 30–50% of children with NF1-OPG experience visual impairment [8] or precocious puberty due to hypothalamic involvement [9].

Since human NF1-OPGs are rarely biopsied and patient-derived xenografts are unavailable, the majority of our insights into pathogenesis of these tumors have derived from the use of genetically-engineered mouse (GEM) strains [10, 11]. These GEM models have been particularly informative for preclinical therapeutic drug discovery and evaluation, resulting in the identification of mechanistic target of rapamycin and MEK as potential targets for optic glioma treatment [12–14], now under evaluation in human clinical trials.

In addition, these GEM models have been useful in defining the potential cells of origin for murine optic glioma. In these studies, somatic *Nf1* loss in BLBP^+^ [15] or GFAP^+^ [11] neuroglial progenitor cells that also co-express the CD133 stem cell marker results in optic glioma formation by 3 months of age. However, somatic *Nf1* loss in NG2^+^ cells during embryogenesis [16] or postnatal GFAP^+^ astrocytes [17] do not generate optic gliomas. In addition, these *Nf1* GEM strains support a regional origin for optic glioma cells of origin to the embryonic third ventricle (TVZ) [17], consistent with one human study using gene expression profiling [18].

The prevalence of Olig2^+^ cells in human gliomas [5], coupled with recent findings that Olig2^+^ oligodendrocyte precursor cells (OPCs) are the cells of origin for murine malignant glioma [19], raises the possibility that OPCs might be another tumor-initiating cell population for *Nf1* murine optic glioma. Herein, we employ two distinct Cre transgenic mouse strains, in which somatic *Nf1* gene inactivation occurs in Olig2^+^ glial progenitor cells or CD133^+^ neural progenitor/stem cells, to demonstrate that the cell of origin is one major determinant in dictating the temporal course of optic gliomagenesis in mice.

## RESULTS AND DISCUSSION

### Mouse optic gliomas harbor increased numbers Olig2^+^ cells

**Figure 1:**
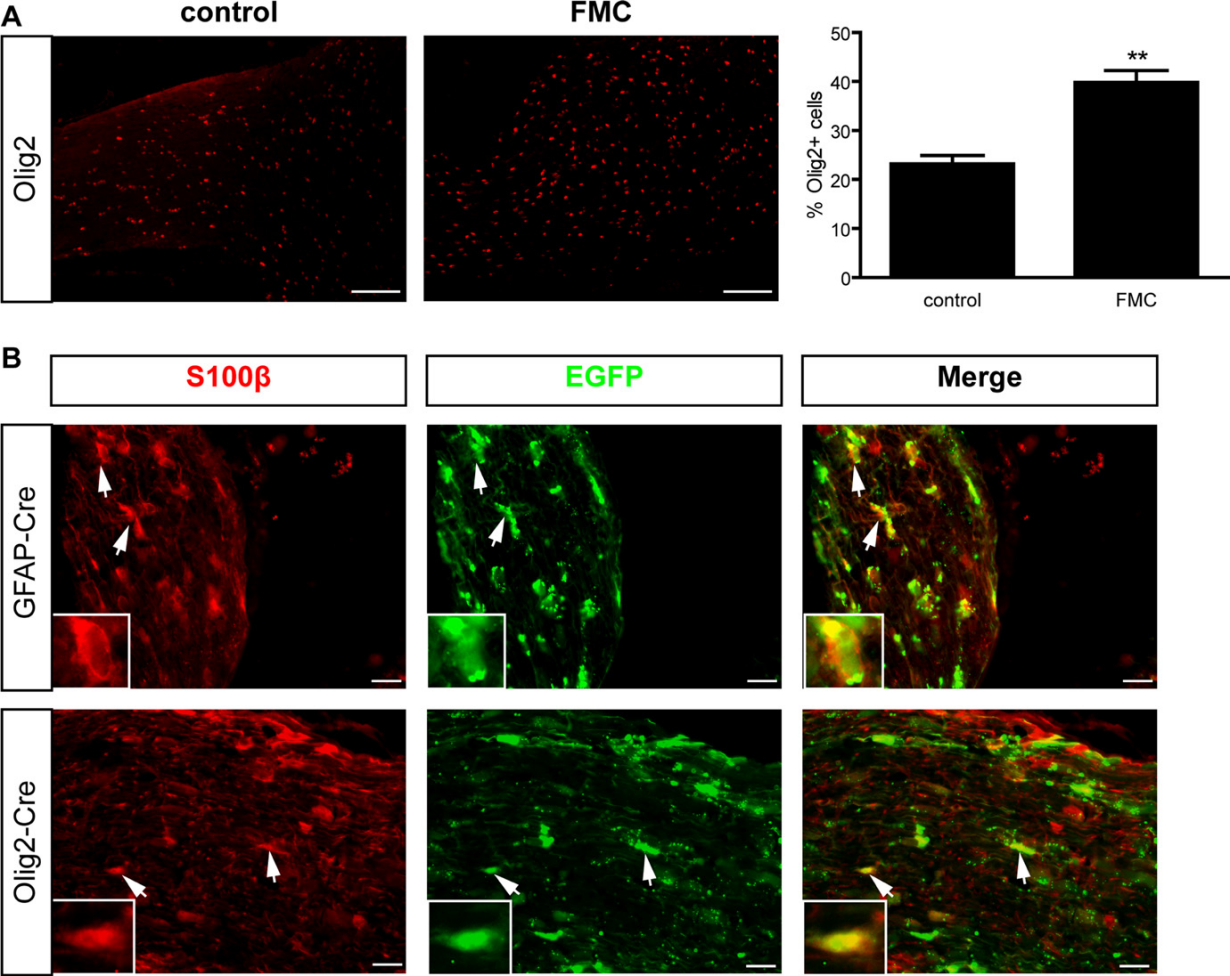
Mouse optic gliomas harbor increased numbers of Olig2^+^ cells (**A**) 3-month-old FMC optic gliomas exhibit a 1.7- fold increase in the percentage of Olig2^+^ cells relative to non-neoplastic optic nerves. Error bars, mean ± SEM. **p = 0.0011. (**B**) Optic nerves from Rosa-GREEN x GFAP-Cre and Olig2-Cre mice, respectively, reveal EGFP^+^/S100β^+^ glia. Representative images include insets of immunopositive cells (arrows). Scale bar, 10 μm.

Previous studies in human low-grade gliomas (PAs) have revealed increased numbers of Olig2^+^ cells [5]. To determine whether these findings were similarly observed in *Nf1* murine optic gliomas, we leveraged a representative GEM strain (*Nf1*^flox/mut^; GFAP-Cre; “FMC”), which harbors a germline inactivating *Nf1* gene mutation, but undergoes somatic loss of the remaining wild-type *Nf1* allele (*Nf1*^flox^ allele) in GFAP-Cre-expressing neuroglial progenitors by embryonic day 14.5 (E14.5). By 3 months of age, ~90% of FMC mice form low-grade gliomas within the prechiasmatic optic nerves and chiasms, characterized by increased proliferation, microglia infiltration, nuclear atypia, cellular pleiomorphism, axonal damage, and retinal ganglion cell death [11]. Similar to their human counterparts, more Olig2^+^ cells (1.7-fold) were found in FMC mouse optic gliomas relative to their non-neoplastic optic nerve counterparts (Figure 1A). Increased Olig2^+^ cell content was also observed in other *Nf1* GEM strains with optic glioma (data not shown).

### Somatic *Nf1* loss in Olig2^+^ cells results in delayed optic glioma formation

To determine whether Olig2^+^ cells can generate astroglial cells in the optic nerve, Olig2-Cre mice were intercrossed with Rosa-GREEN reporter mice. The Olig2-Cre mouse employed is a transgenic strain containing endogenous *Olig2* promoter elements, thus fully recapitulating normal Olig2 expression *in vivo* [20]. Similar to the GFAP-Cre mice used to generate FMC optic gliomas, Olig2-Cre mice also give rise to S100β^+^ glia (Figure 1B) and GFAP^+^ astrocytes (data not shown). Since Olig2^+^ cells can give rise to astrocytes in the optic nerve, we next sought to determine whether *Nf1* loss in Olig2^+^ cells was sufficient for optic glioma formation in *Nf1*+/− mice.

**Figure 2:**
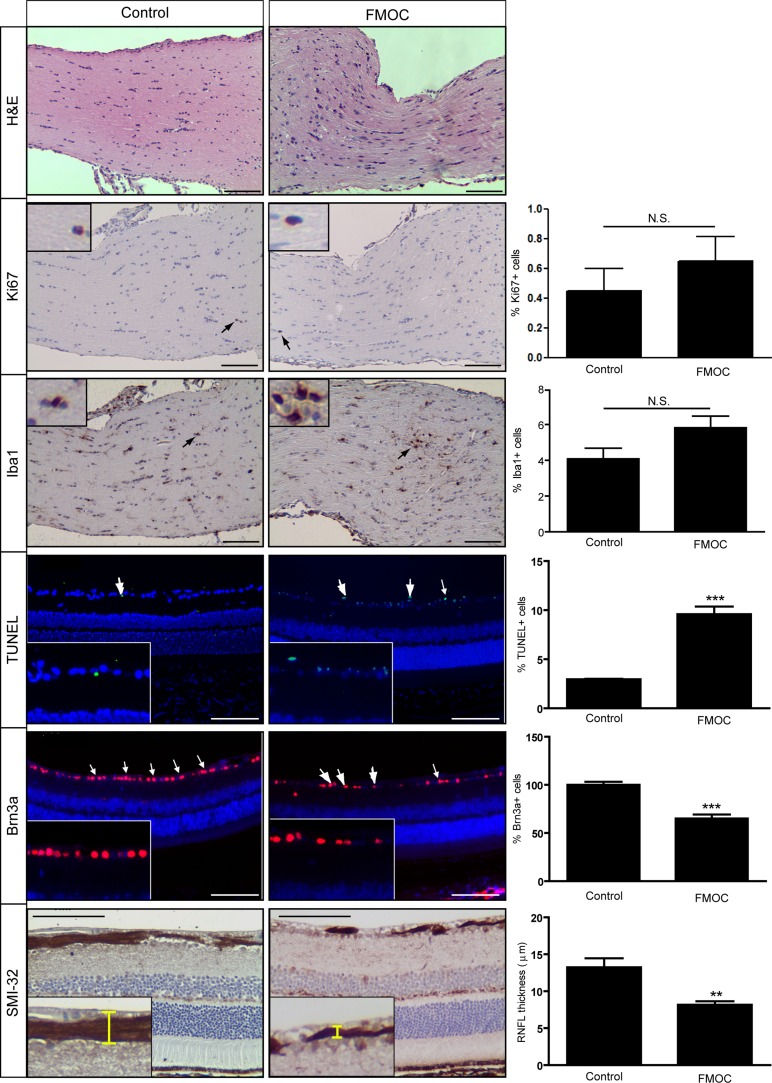
FMOC mice do not develop optic gliomas at 3 months of age FMOC mice have increased cellularity (hematoxylin/eosin) compared to controls (*n* = 5 mice/group). No change in the percentage of Ki67^+^ or Iba1^+^ cells was observed. There was increased retinal cell apoptosis (%TUNEL^+^ cells), fewer Brn3a^+^ cells, and reduced retinal nerve fiber layer (RNFL) thickness in FMOC mice relative to controls. Nuclei were counterstained with DAPI (blue). Representative images include insets of immunopositive cells (arrows). Scale bar, 100 μm. Error bars, mean ± SEM. ***p < 0.0003; **p = 0.0078; N.S. = not significant

While *Nf1*^flox/mut^; Olig2-Cre (“FMOC”) mice had larger optic nerve volumes relative to control littermates at 3 months of age (Supplementary Figure 1A), in striking contrast to FMC optic nerves, there was no evidence for glioma. In this respect, increased Iba1^+^ microglia and proliferation (Ki67^+^ cells) were not observed relative to control littermates (Figure 2). While no tumors were identified, there were more enlarged axons (Supplementary Figure 1B), increased myelin sheath thickness (Supplementary Figure 1C), and reduced g-ratios (Supplementary Figure 1D), similar to that observed following *Nf1* loss in other oligodendrocyte lineage cells [21], as well as associated retinal pathology, including increased TUNEL^+^ labeling, decreased Brn3a^+^ retinal ganglion cells, and thinning of the retinal nerve fiber layer (Figure 2).

We next sought to determine whether somatic *Nf1* loss in Olig2^+^ progenitors delayed the onset of glioma development. For this analysis, we chose to examine these mice at both 4.5 and 6 months of age. At 4.5 months of age, FMOC mice had enlarged optic nerve volumes (0.088mm^3^ ± 0.016), but microglia content (%Iba1^+^ cells; 6.92 ± 0.5) and proliferation (%Ki67^+^ cells; 0.28 ± 0.35) were comparable to that observed in control mice. These results suggest that optic gliomas had not formed at this time point.

**Figure 3:**
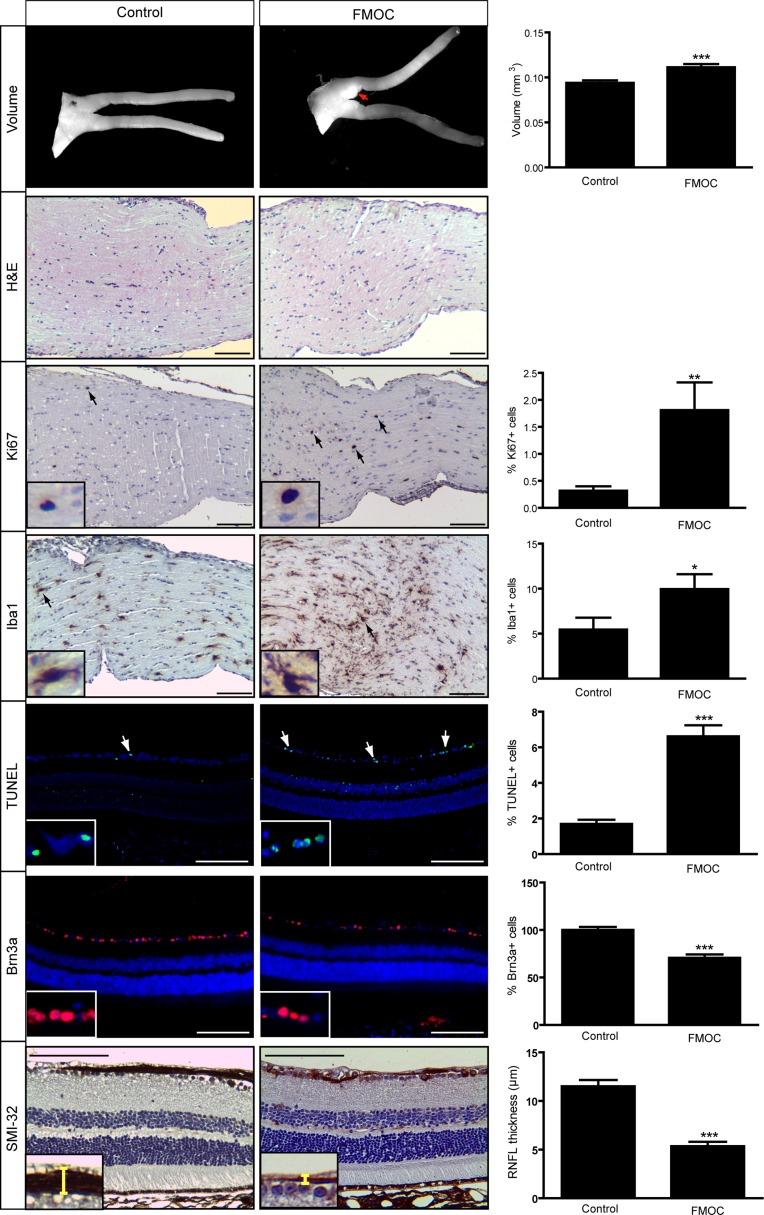
FMOC mice develop optic glioma by 6 months of age FMOC mice have larger optic nerve volumes relative to controls as well as increased cellularity (hematoxylin/eosin) (*n* = 5 mice/group). 6-month-old FMOC mice show increased percentages of Ki67^+^ and Iba1^+^ cells. There was also increased retinal cell apoptosis (%TUNEL^+^ cells), fewer Brn3a^+^ cells, and reduced RNFL thickness in FMOC mice. Nuclei were counterstained with DAPI (blue). Representative images include insets of immunopositive cells (arrows). Scale bar, 100 μm. Error bars, mean ± SEM. ***p < 0.0006; **p = 0.0038; *p = 0.049.

However, at 6 months of age, optic nerve volumes in FMOC mice were increased (Figure 3), with enlarged axons, increased myelin sheath thickness (Supplementary Figure 2), and retinal pathology (Figure 3). Importantly, there was now a 1.8-fold increase in the percent of Iba1^+^ microglia and a 5.7-fold increase in the percent of Ki67^+^ cells relative to controls (Figure 3), indicating glioma. In addition, there was increased retinal apoptosis (%TUNEL^+^ cells), retinal ganglion cell loss (%Brn3a^+^ cells), and retinal nerve fiber layer thinning (SMI-32 immunostaining). Collectively, these data reveal that while somatic *Nf1* loss in Olig2^+^ cells results in optic glioma formation, the latency is extended relative to FMC mice [11].

### Delayed optic gliomagenesis reflects distinct cells of origin

**Figure 4:**
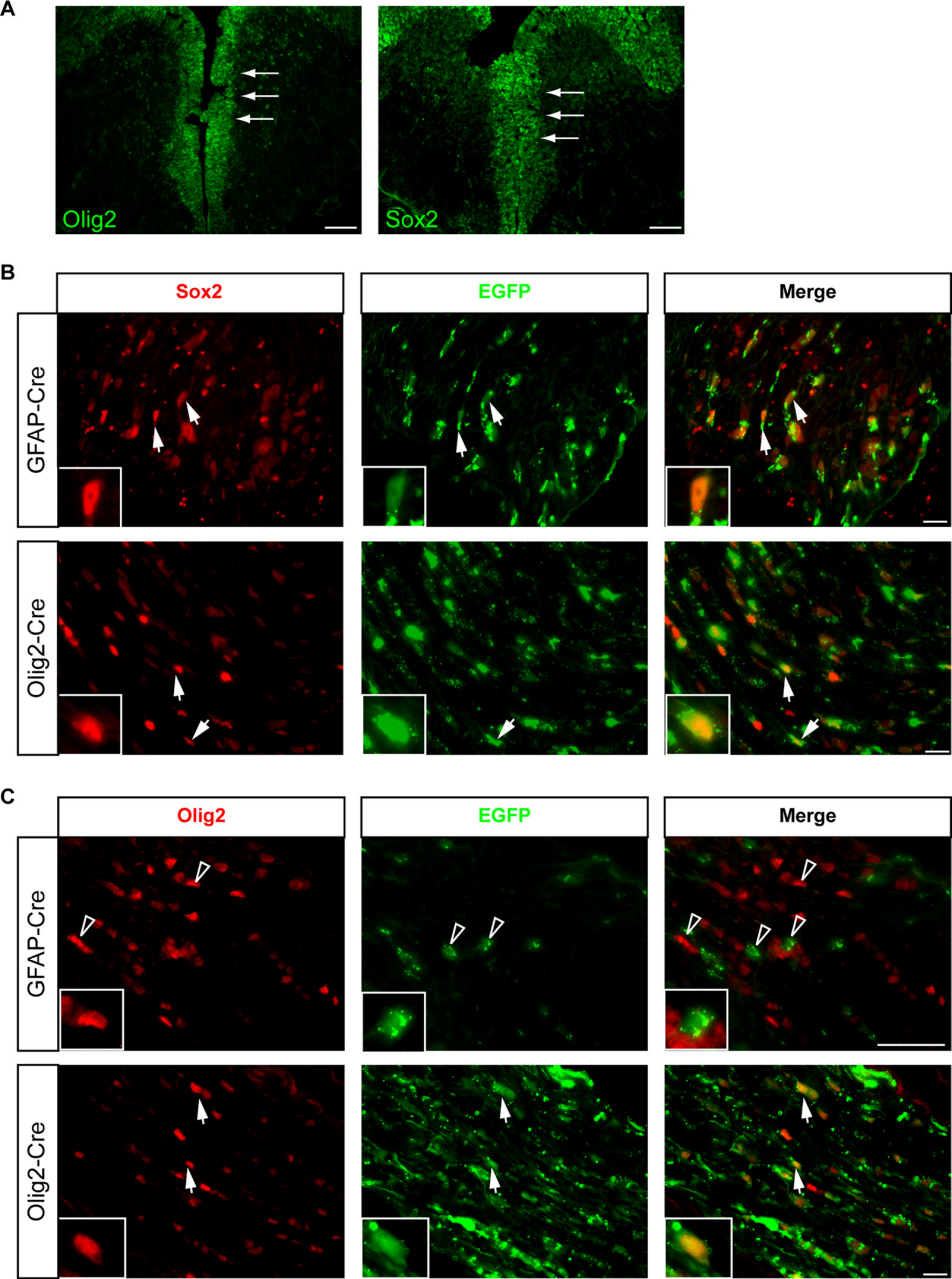
Cells of origin for FMOC optic gliomas are distinct from those that give rise to FMC mouse optic gliomas (**A**) Olig2^+^ cells line the third ventricular surface as early as E11.5. Sox2 expressing cells are located in the same region within the third ventricle at E11.5 as Olig2^+^ cells. (**B**) Optic nerve sections from Rosa-GREEN x GFAP-Cre and Olig2-Cre mice at 3 weeks of age, respectively, contain both EGFP/Sox2 double-positive cells. (**C**) In contrast, EGFP/Olig2^+^ cells are only found in the optic nerves of Rosa-GREEN x Olig2-Cre mice, but not in those from Rosa-GREEN x GFAP-Cre mice, at 3 weeks of age. Representative images are shown with insets of immunopositive cells. Arrowheads indicate single positive cells, whereas arrows indicate double-positive cells.

One possible interpretation for the delay in glioma formation is the timing or brain location of somatic *Nf1* loss in FMOC mice relative to FMC mice. However, the Olig2-Cre transgene was expressed by E12, similar to previously-employed *Nf1* optic glioma GEM models [15], labeling cells along the lateral (data not shown) and third ventricles where Sox2^+^ progenitors reside (Figure 4A).

Next, we performed lineage tracing of the cells of origin in these mouse strains, leveraging Rosa-GREEN reporter mice intercrossed with GFAP-Cre and Olig2-Cre mice, respectively. Within the optic nerves both GFAP-Cre x Rosa-GREEN and Olig2-Cre x Rosa-GREEN mice contained EGFP^+^ cells that co-labeled with S100β (Figure 1C) or Sox2 (Figure 4B). In contrast, EGFP/Olig2^+^ cells were only found in the optic nerves of Olig2-Cre x Rosa-GREEN mice (Figure 4C). The fact that the GFAP-Cre transgene does not generate Olig2^+^ cells suggests that the cells of origin for FMOC optic gliomas are distinct from those that give rise to FMC optic gliomas.

Finally, to demonstrate that the cell of origin is a major determinant underlying the observed differences in tumor latency, we employed a Cre transgenic strain in which *Nf1* loss could be specifically induced in CD133^+^ neural progenitor/stem cells at a similar time as occurs in FMC mice. The prominin1-Cre^ER^ strain contains a tamoxifen-regulated Cre recombinase protein expressed from the endogenous prominin-1 (CD133) promoter [22], thus targeting neural stem cells in the brain [23] expressing GFAP [24]. As such, optic nerves from Prom1-Cre^ER^ x Rosa-GREEN mice injected with tamoxifen and progesterone at E15 contained EGFP^+^ cells that co-labeled with S100β and Sox2, but not Olig2, similar to that observed with GFAP-Cre x Rosa-GREEN mice (data not shown).

**Figure 5:**
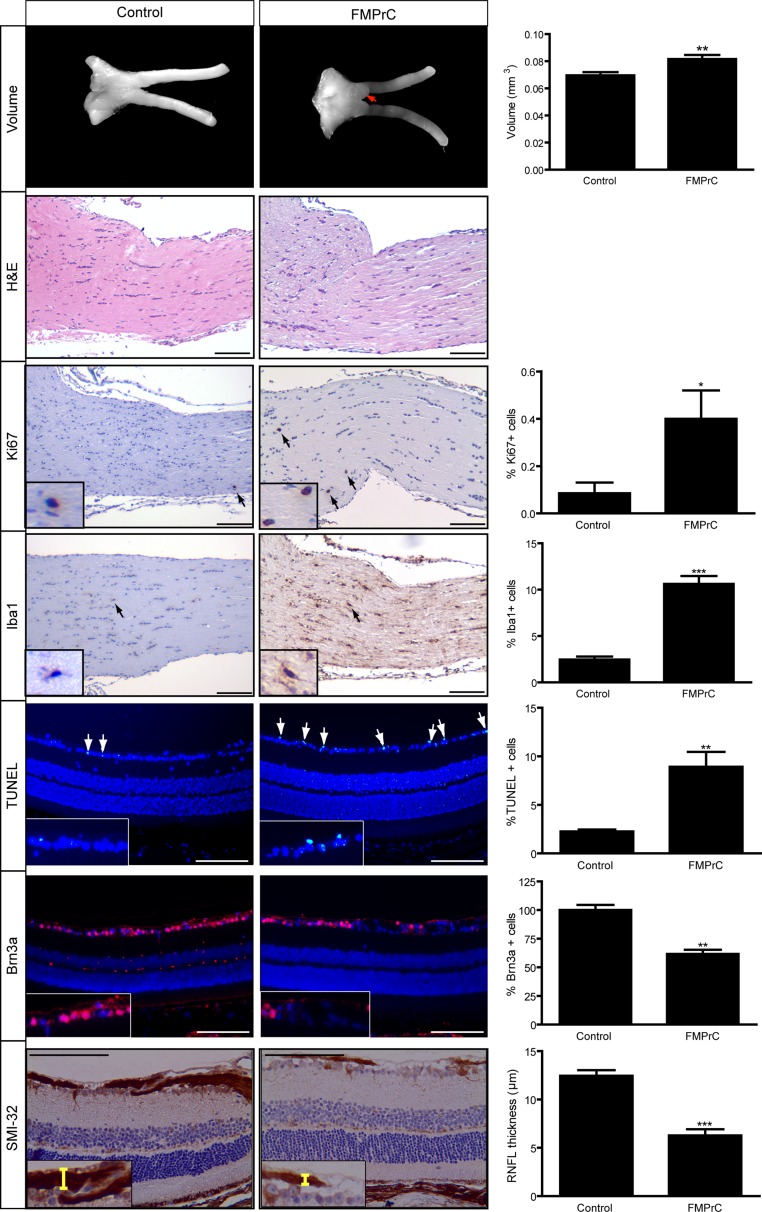
Somatic *Nf1* loss in neural stem cells recapitulates the temporal course of optic gliomagenesis observed in FMC mice FMPrC mice have larger optic nerve volumes relative to controls, as well as increased cellularity (hematoxylin/eosin) (*n* = 5 mice/group). 3-month-old FMPrC mice show increased percentages of Ki67^+^ and Iba1^+^ cells. There was also increased retinal cell apoptosis (%TUNEL^+^ cells), fewer Brn3a^+^ cells, and reduced RNFL thickness in FMPrC mice. Nuclei were counterstained with DAPI (blue). Representative images include insets of immunopositive cells (arrows). Scale bar, 100 μm. Error bars, mean ± SEM. ***p < 0.0003; **p = 0.0079; *p = 0.0429.

Based on these findings, we generated *Nf1*^flox/mut^; Prom1-Cre^ER^ (FMPrC) mice for tamoxifen and progesterone injection at E15. At 3 months of age, FMPrC mice had increased optic nerve volumes, percentages of Iba1^+^ cells, and percentages of Ki67^+^ cells (Figure 5), similar to that observed with FMC mice. In addition, these mice had clear retinal pathology, with increased retinal cell apoptosis (%TUNEL^+^ cells), reduced retinal ganglion cell numbers (%Brn3a^+^ cells), and decreased retinal nerve fiber layer thickness (SMI-32 immunostaining). These results indicate that somatic *Nf1* inactivation in neural progenitor/stem cells (GFAP^+^ or CD133^+^ cells) results in gliomagenesis at 3 months of age, whereas *Nf1* loss in Olig2^+^cells generates tumors with a prolonged latency. While the cell lineage tracing experiments using Rosa-GREEN strains demonstrate that Olig2^+^ cells in the optic nerve do not arise from CD133^+^ or GFAP-Cre transgene-expressing cells at E15–16, future studies will be required to identify the specific subpopulations of progenitors cells capable of serving as the potential cells of origin for these tumors.

### The impact of the cell of origin on the temporal course of gliomagenesis

Taken together with our previous studies showing that *Nf1+/*− mice with somatic *Nf1* loss in NG2^+^ cells do not form optic gliomas [16], we now conclude that both neuroglial progenitor cells (GFAP^+^, BLBP^+^, CD133^+^ cells) and pre-OPCs (Olig2^+^, NG2^neg^ cells) can serve as initiating cells for murine *Nf1* low-grade glioma, but with different latencies to tumor formation [25]. As such, *Nf1+/*− mice with somatic *Nf1* inactivation in GFAP-Cre^+^ [11], BLBP^+^ [15], or CD133^+^ neural stem cells all develop tumors by 3 months of age, whereas those with *Nf1* inactivation in pre-OPCs form gliomas later. Since neither GFAP-Cre nor prominin-1-Cre^ER^ mice treated with tamoxifen at E15 generate Olig2^+^ cells, yet all Cre driver lines give rise to GFAP^+^ and S100β^+^ macroglial cells, we propose that Olig2^+^ cells represent a glial precursor lineage distinct from those delineated by GFAP-Cre- or prominin-1-Cre^ER^-mediated somatic *Nf1* loss. These findings suggest that there are multiple distinct cells of origin for glioma that each can generate astrocytes through unique differentiation pathways. Consistent with this hypothesis, whole-tumor RNA-sequencing analyses of FMC and FMOC optic gliomas demonstrates that these two brain tumors are molecularly-distinct entities [26].

While the studies herein are novel with respect to low-grade glioma, they join a growing body of evidence that suggests that the cell of origin is critical for creating the diversity of human cancers. As such, whereas luminal and basal cells can both serve as cells of origin for prostate cancer, each generates distinct subtypes with different histological and molecular features [27]. Similarly, CD133-negative luminal cells give rise to highly metastatic breast cancers, but CD133-positive luminal cells generate both basal and luminal tumors with little metastatic potential [28]. Finally, dorsal brainstem progenitor cells are the cells of origin for one subtype of medulloblastoma, while cerebellar granule precursors are responsible for a different subtype of this common pediatric brain tumor, each with different molecular properties [29]. Although these reports highlight the ways in which the cell of origin creates molecular tumor diversity, our findings demonstrate that histologically-similar tumors can be generated with different latencies based entirely on their progenitor cell origins. Future studies aimed at characterizing the different responsible progenitor cell populations may yield important insights into the diversity of cellular origins of pediatric glioma relevant to the clinical spectrum of OPGs encountered in children with NF1.

## MATERIALS AND METHODS

### Mice

*Nf1+/*− mice [30] were bred with *Nf1*^f/wt^ mice to produce *Nf1*^flox/mut^ mice, and intercrossed with *Nf1*^flox/flox^*;* Olig2-Cre mice to generate *Nf1*^flox/mut^*;* Olig2-Cre (FMOC) mice, while *Nf1*^flox/mut^*;* GFAP-Cre (FMC; [11]) and *Nf1*^flox/flox^ [31] control mice were established as previously reported. *Nf1*^flox/mut^ mice were also intercrossed with *Nf1*^flox/flox^*;* Prom1-Cre^ER^ mice [22] to generate *Nf1*^flox/mut^*;* Prom1-Cre^ER^ (FMPrC) mice. Timed-pregnant FMPrC dams (gestational day 14–16) underwent oral gavage with 1mg tamoxifen (Sigma-Aldrich, St. Louis, MO) and 1 mg progesterone (Sigma-Aldrich, St. Louis, MO) in 100μl of corn oil suspension. Olig2-Cre mice (B6-Olig2^tm2 (TVA, cre)Rth^/J; Jackson Laboratory; [20]) and GFAP-Cre mice [32] were intercrossed with Rosa-GREEN mice (B6.Cg-*Gt(ROSA)26Sor*^tm(CAGZsGreen1)Hze+^*/*J; Jackson Laboratory) for cell fate mapping. Mice were backcrossed and maintained on a C57Bl/6 background and used in accordance with approved Animal Studies protocols. The recombination frequency of Olig2-Cre (11.11%; data not shown) and GFAP-Cre (13.36%; [16]) mice were determined following intercrossing with Rosa-GREEN mice.

### Immunohistochemistry

Paraffin and frozen section immunohistochemical analyses were performed [33] using specific antibodies (Supplementary Table 1), followed by either Alexa-Fluor-tagged secondary antibodies (Invitrogen, Grand Island, NY) and DAPI counterstaining or biotinylated secondary antibodies and Vectastain Elite ABC development. Terminal deoxynucleotide transferase-mediated dUTP nick-end labeling (TUNEL) staining was performed as before [33].

### Optic nerve measurements

Optic nerves with an intact chiasm were microdissected, photographed, and optic nerve volumes calculated [13].

### Statistical analysis

Statistical analysis was performed as previously described [33].

## SUPPLEMENTARY MATERIALS FIGURES AND TABLES


