# Supplementary material for: The cell of origin dictates the temporal course of neurofibromatosis-1 (Nf1) low-grade glioma formation
Source: Oncotarget. 2017 May 3;8(29):47206–15. doi: 10.18632/oncotarget.17589 (PMC5564557; doi:10.18632/oncotarget.17589)
Supplement: Supplementary file 1 [file oncotarget-08-47206-s001.pdf]

The cell of origin dictates the temporal course of neurofibromatosis-1 (*Nf1*) low-grade glioma formation

Supplementary Materials

MATERIALS AND METHODS

Morphometry of optic nerve axons

Mice were perfused with Ringer's solution followed by 2% paraformaldehyde and 2.5% glutaraldehyde in 0.3M cacodylate buffer (pH 7.4). Both retrobulbar optic nerve segments from each mouse were post-fixed in 1% buffered OsO_4, successively dehydrated in ethanol/acetone, and embedded in Durcupan (Electron Microscopy Sciences, Hatfield, PA) for light and electron microscopy. Transverse semi-thin sections (0.2 µm) obtained at a 1-mm distance behind the eyeballs were stained with paraphenylenediamine (1% in isopropanol/methanol; 1 hour) to optimize contrast of the myelinated fibers for automated axon counts. Mosaic images of paraphenylenediamine-stained nerve cross-sections were produced on an Olympus BX51WI Microscope at 100x equipped with a mechanical stage and Microbrightfield Neurolucida software and captured with an Optronics Microfire (1600×1200) CCD camera (0.073 µm/pixel). Post-processing, segmentation and size measurements were performed using Image J. The measurements excluded the myelin sheath and used eccentricity and size criteria to eliminate glial and vascular profiles. Axon sizes (areas) were mapped into bins to assess size distributions of the axonal populations by area. Axonal profiles were also examined by transmission electron microscopy (FEI-Spirit) in 50nm sections obtained from selected specimens in the prechiasmatic region.

G-ratio calculations

The g-ratio was calculated on 100 nm-thin orthogonal sections acquired at 1100x magnification by transmission electron microscopy. More than 100 axons were randomly selected, and the g-ratio determined by calculating the square root of the ratio of the axon orthogonal section surface area excluding the myelin sheath [A1] by the axon orthogonal section surface area including the myelin sheath [A2] (Supplementary Figure 1D).

Supplementary Table 1: Antibodies

Antibody	Cell type	Species	Dilution	Antigen retrieval solution	Source
Brn3a	RGC	mouse, monoclonal	1:500	0.1M citric acid + 0.1M sodium citrate	Santa Cruz
Iba1	microglia	rabbit, polyclonal	1:1,000	0.1M citric acid + 0.1M sodium citrate	Wako
Ki-67	proliferating cells	mouse, monoclonal	1:500	0.1M citric acid + 0.1M sodium citrate	BD Pharmingen
Olig2	oligodendrocyte precursor cells	rabbit, polyclonal	1:10,000	0.1M citric acid + 0.1M sodium citrate	Charles D. Stiles (Boston, MA)
S100β	glia	mouse, monoclonal	1:100	0.1M citric acid + 0.1M sodium citrate	Abcam
SMI32		mouse, monoclonal	1:500	0.1M citric acid + 0.1M sodium citrate	Biolegend
Sox2	stem cells	mouse, monoclonal	1:500	N/A	Abcam

N/A = not applicable

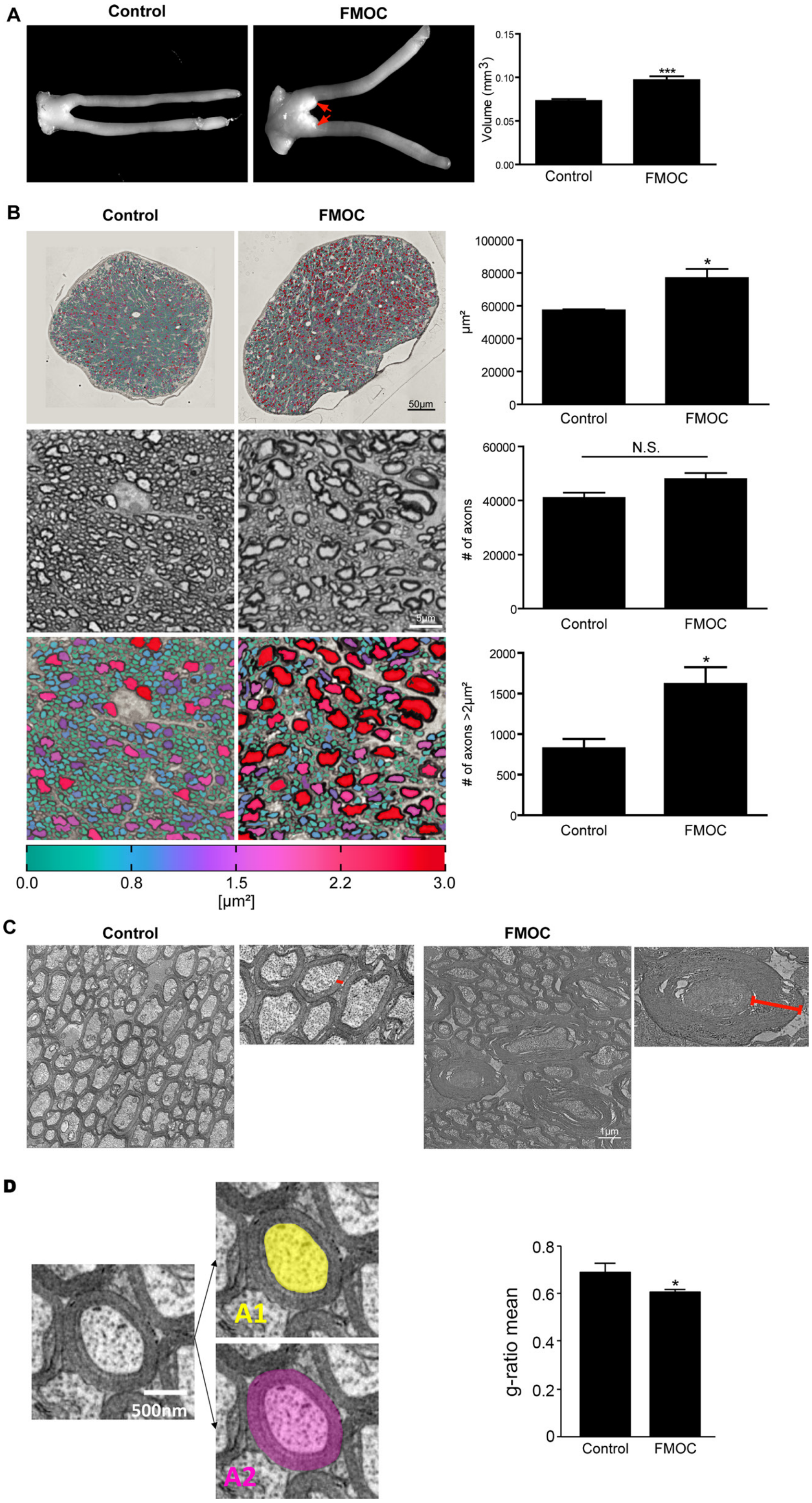

Supplementary Figure 1: Optic nerve pathology at 3 months of age. (**A**) FMOC mice have larger optic nerve volumes relative to control mice at 3 months of age. Representative images of the optic nerves from control and FMOC mice are shown (*n* = 10 mice/group). (**B**) Cross-sectional photomicrographs demonstrate increased numbers of large caliber axons in FMOC mice at 3 months of age. Automated total axon counts and size determinations were obtained from high-resolution (100x) mosaic images of paraphenylene-diamine-stained, semi-thin sections. No changes in the numbers of axons were observed at 3 months of age. The distribution of axon diameters is shifted in FMOC optic nerves, resulting in an increase in the number of larger axons (> 2 µm2). Axons are colored according to their size as reference, with red coloring axons superior or equal to 3µm². Error bars represent mean ± SEM. Asterisks denote statistically significant differences ***$p < 0.0001$; *$p = 0.03$, N.S. = not significant. (**C**) Electron micrographs of the enlarged optic nerve axon myelin sheaths from FMOC mice are shown relative to Olig2-Cre controls. Representative images are shown. (**D**) FMOC mice have reduced mean g-ratios in the optic nerves relative to control mice at 3 months of age (*n* = 3 mice/group; $P = 0.02$).

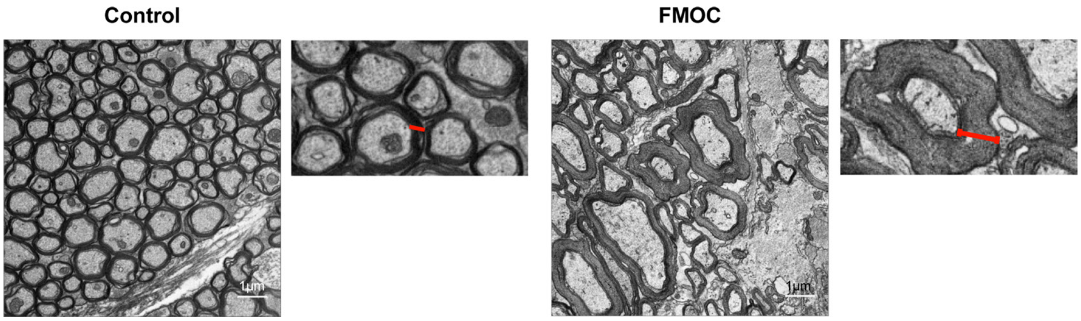

Supplementary Figure 2: Optic nerve pathology at 6 months of age. Electron micrographs of the optic nerve axon myelin sheaths are enlarged in FMOC mice relative to Olig2-Cre controls. Representative images are shown.
